# Supplementary material for: Deletion of wfs1 Impairs Oligodendrocyte Precursor Cells Dorsal Distribution and Myelination Through the wfs1-hmgcs1 Axis in Zebrafish
Source: Biology (Basel). 2026 Aug 16;15(16):1402. doi: 10.3390/biology15161402 (PMC13510151; doi:10.3390/biology15161402)
Supplement: Supplementary file 1 [file biology-15-01402-s001.zip › File S1-Transcriptome sequencing data and differentially expressed genes (DEGs) in wfs1-- zebrafish at 5 dpf/2.KEGG/wfs1KOvsWT/ALL/src/dre00760.html]

dre00760


- dre:402953

- Up regulated genes

402953\_1(1.71924648864672)

- dre:572070

- Up regulated genes

110438097(4.95899171594655)

- dre:572070

- Up regulated genes

110438097(4.95899171594655)

- dre:572070

- Up regulated genes

110438097(4.95899171594655)

- dre:572070

- Up regulated genes

110438097(4.95899171594655)

- dre:402953

- Up regulated genes

402953\_1(1.71924648864672)

- dre:101886891

- Up regulated genes

101886891(1.44936351432932)

- dre:101886891

- Up regulated genes

101886891(1.44936351432932)

Close
